# An Alcohol Dehydrogenase Gene from *Synechocystis* sp. Confers Salt Tolerance in Transgenic Tobacco

**DOI:** 10.3389/fpls.2017.01965

**Published:** 2017-11-17

**Authors:** So Young Yi, Seong Sub Ku, Hee-Jung Sim, Sang-Kyu Kim, Ji Hyun Park, Jae Il Lyu, Eun Jin So, So Yeon Choi, Jonghyun Kim, Myung Suk Ahn, Suk Weon Kim, Hyunwoo Park, Won Joong Jeong, Yong Pyo Lim, Sung Ran Min, Jang Ryol Liu

**Affiliations:** ^1^Plant Systems Engineering Research Center, Korea Research Institute of Bioscience and Biotechnology, Daejeon, South Korea; ^2^Institute of Agricultural Science, Chungnam National University, Daejeon, South Korea; ^3^Center for Genome Engineering, Institute for Basic Science, Daejeon, South Korea; ^4^Biological Resources Center, Korea Research Institute of Bioscience and Biotechnology, Daejeon, South Korea; ^5^Department of Horticulture, Chungnam National University, Daejeon, South Korea

**Keywords:** alcohol dehydrogenase, cyanobacteria, green leaf volatiles (GLVs), *Z*-3-hexenol, priming, salt tolerance

## Abstract

*Synechocystis* salt-responsive gene 1 (*sysr1*) was engineered for expression in higher plants, and gene construction was stably incorporated into tobacco plants. We investigated the role of Sysr1 [a member of the alcohol dehydrogenase (ADH) superfamily] by examining the salt tolerance of *sysr1*-overexpressing (*sysr1*-OX) tobacco plants using quantitative real-time polymerase chain reactions, gas chromatography-mass spectrometry, and bioassays. The *sysr1*-OX plants exhibited considerably increased ADH activity and tolerance to salt stress conditions. Additionally, the expression levels of several stress-responsive genes were upregulated. Moreover, airborne signals from salt-stressed *sysr1*-OX plants triggered salinity tolerance in neighboring wild-type (WT) plants. Therefore, Sysr1 enhanced the interconversion of aldehydes to alcohols, and this occurrence might affect the quality of green leaf volatiles (GLVs) in *sysr1*-OX plants. Actually, the *Z*-3-hexenol level was approximately twofold higher in *sysr1*-OX plants than in WT plants within 1–2 h of wounding. Furthermore, analyses of WT plants treated with vaporized GLVs indicated that *Z*-3-hexenol was a stronger inducer of stress-related gene expression and salt tolerance than *E*-2-hexenal. The results of the study suggested that increased C_6_ alcohol (*Z*-3-hexenol) induced the expression of resistance genes, thereby enhancing salt tolerance of transgenic plants. Our results revealed a role for ADH in salinity stress responses, and the results provided a genetic engineering strategy that could improve the salt tolerance of crops.

## Introduction

Alcohol dehydrogenases (ADHs, alcohol: NAD^+^ oxidoreductase, EC 1.1.1.1) belong to the dehydrogenase enzyme superfamily, and they are widely distributed across all organism types (Chase, 1999; Jornvall et al., 2010; Strommer, 2011; Alka et al., 2013). These enzymes catalyze the interconversion between alcohols and aldehydes (Hoog et al., 2003; Thompson et al., 2007). ADHs are classified into three main superfamilies based on the amino acid sequence length, namely medium- (approximately 350 amino acids), short- (approximately 250 amino acids), and long-chain (600–750 residues) ADHs (Chase, 1999; Alka et al., 2013; Jornvall et al., 2013). Most plant ADHs, characterized at the gene level, belong to the medium-chain ADH protein superfamily (Chase, 1999).

The expression of *ADH* genes generally produces enzymes that are not only active when plants are exposed to various stresses, but also during all plant growth stages under normal conditions. ADHs help protect plants from the effects of hypoxic stress induced by flooding (Kennedy et al., 1992; Bailey-Serres and Voesenek, 2008), and the enzymes also have functions associated with seed development (Hanson et al., 1984; MacNicol and Jacobsen, 2001) and aerobic metabolism in pollen grains (Bucher et al., 1995). *ADH1* expression is induced by various environmental stresses, including cold and osmotic stresses (Christie et al., 1991; Conley et al., 1999), wounding (Kato-Noguchi, 2001), and drought stress (Dolferus et al., 1994; Senthil-Kumar et al., 2010). The *ADH1* expression level is also upregulated in response to the application of exogenous abscisic acid (de Bruxelles et al., 1996), and salinity stress induces the accumulation of *ADH* mRNA in soybeans, grass peas, and *Arabidopsis* (Manak et al., 2002; Sobhanian et al., 2010; Chattopadhyay et al., 2011). However, very little is known about the effects of ADHs on plant physiology during exposure to abiotic stress conditions.

Alcohol dehydrogenase activity is directly and indirectly involved in the production of green leaf volatiles (GLVs), as suggested by the lack an aroma in the crushed leaves of *adh* mutant plants (Salas et al., 2005). GLVs include aldehydes, esters, and alcohols of six-carbon compounds that are released after wounding (Matsui, 2006). GLVs originate in the hydroperoxide lyase (HPL) branch of the oxylipin pathway, and they are formed from fatty acids (Matsui, 2006). ADHs help catalyze the interconversion of C_6_ volatiles (e.g., hexenal to hexenol and *Z*-3-hexenal to *Z*-3-hexenol) (Bicsak et al., 1982; Longhurst et al., 1990). Almost all plants produce GLVs, and their release can be caused by abiotic stimuli (Tingey et al., 1980; Loreto and Delfine, 2000; Gomi et al., 2003; Vallat et al., 2005; Teuber et al., 2008; Brilli et al., 2011), herbivores (Turlings et al., 1995; Heil and Silva Bueno, 2007), or pathogens (Croft et al., 1993; Shiojiri et al., 2006). Moreover, plants release GLVs almost immediately after their cellular structures are damaged (Behnke et al., 2009). For example, *Arabidopsis thaliana* leaves produce *Z*-3-hexenal 30–45 s after being wounded, and *Z*-3-hexenol and hexenyl acetate are released after approximately 5 min (D’Auria et al., 2007). Physiologically, GLVs function as signaling molecules that induce plant defense responses (Frost et al., 2007). The treatment of plants with GLVs induces the production of downstream metabolites, and it increases the expression of defense-related genes (Bate and Rothstein, 1998; Farag et al., 2005). The release of GLVs in response to insect feeding is thought to prime neighboring plants for potential damage from herbivory (Engelberth et al., 2004), and primed plants display quicker and more powerful defense responses when challenged by biotic and abiotic stresses (Conrath, 2009).

Plants show various responses to salt stress that enable them to tolerate adverse conditions. In response to high salt conditions, the expression levels of several genes are upregulated, and the encoded proteins directly or indirectly contribute to plant protection (Winicov, 1998). There are numerous candidate genes that could be used to transform crops to improve salinity tolerance, and genes that increase salt tolerance can be divided into three groups. The first group includes genes that control salt transport. For example, the overexpression of *SOS1*, which encodes a plasma membrane Na^+^/H^+^ antiporter, increases the salinity tolerance of transgenic *Arabidopsis* (Shi et al., 2003). The second group consists of genes that have an osmotic or protective function. As shown in a previous study, the overexpression of mannitol-1-phosphate dehydrogenase (*mt1D*), which mediates mannitol synthesis in bacteria, can increase salinity tolerance in wheat (Abebe et al., 2003). The third group includes genes that mediate the detection, signaling, and regulatory pathways involved in global salinity tolerance. Improving crop salt tolerance by overexpressing transcription factor genes has been described in model species such as *A. thaliana* (Kasuga et al., 1999; Zhou et al., 2009; Mao et al., 2011; Yang et al., 2011), and it has been demonstrated to a lesser extent in crops such as rice, wheat, tomato, and alfalfa (Roy et al., 2014).

In this study, we incorporated the *Synechocystis* sp. PCC 6906 *sysr1* gene into the *Nicotiana benthamiana* genome. This gene encodes an ADH that catalyzes the reduction of aldehydes to a greater degree than the oxidation of alcohols in *Synechocystis* sp. The results indicated that transgenic *N. benthamiana* plants overexpressing *sysr1* exhibited enhanced salt tolerance. Thus, our data revealed a novel role for *sysr1* in salt-stress responses.

## Materials and Methods

### Plant Materials and Growth Conditions

*Nicotiana benthamiana* plants were grown in a growth chamber set at 25 ± 1°C with a 16-h light (70 μmol m^-2^ s^-1^):8-h dark photoperiod. To generate 35S::*sysr1*-transgenic plants, the *sysr1* coding region was amplified via polymerase chain reactions (PCR) with a forward and reverse primer set (5′-AACACGGGGGACTCTAGAATGATTAACGCCTACGCGGCCC-3′ and 5′-TCGGGGAAATTCGAGCTCTCAATGGCTTAAAACCACACGGT-3′). The amplified fragments were cloned into the *Xba*I/*Sac*I restriction enzyme sites of pHC30 (**Supplementary Figure S2**), which was modified from pCAMBIA3300. The resulting pHC30 vector was used to transform *N. benthamiana* plants, and putative transformants were transferred to soil. DNA isolated from young leaves was used to detect the presence of the transgene via quantitative real-time (qRT)-PCR. Seven independent transgenic lines were established (T_1_: Lines 1, 4, and 6–10).

### Quantitative Real-Time Polymerase Chain Reaction Analysis

Total RNA was isolated from the collected seedlings using an RNeasy mini kit (Qiagen). Approximately 1 μg DNA-free RNA was used for first-strand cDNA synthesis with M-MuLV reverse transcriptase (Enzynomics). qRT-PCR was conducted using the CFX96 qPCR system (Bio-Rad^1^) and SYBR Premix Ex Taq (TaKaRa^2^), and primers (0.1 μM) were used in a 25-μL final volume. The qRT-PCR protocol was as follows: 95°C for 10 min; 40 cycles of 95°C for 5 s and 60 °C for 20 s. A dissociation curve was subsequently generated. All reactions were completed in triplicate, and details about the qRT-PCR primers are provided in Supplementary Table S1.

### Salt-Stress Assay with Transgenic *N. benthamiana* Plants

Homozygous T_3_ *sysr1-*transgenic *N. benthamiana* plants (Lines 1, 4, and 7) were analyzed in a salt-stress assay. The seeds of wild-type (WT) and transgenic plants were surface-sterilized and vernalized at 4 °C for 3 days. Samples were then placed in Petri dishes containing Murashige and Skoog (MS) medium (pH 5.7) supplemented with vitamins, 3% sucrose, and 0.4% (w/v) Phytagel. The seeds were incubated at 25 ± 1°C in an illuminated growth chamber. After 2 weeks, the seedlings were transferred to square Petri dishes containing MS agar (0.6% Phyto Agar) medium, which was supplemented with 300 mM NaCl or 400 mM mannitol for salt-stress treatments. After a 4-week incubation, root lengths, numbers of lateral roots, and fresh weights were recorded.

Salt tolerance at the adult stage was evaluated according to the method of Sun et al. (2013). WT and *sysr1*-overexpressing (*sysr1*-OX) plants grown on MS agar medium for 4 weeks were transferred to soil, and the samples were then acclimated for 2 weeks. Each plant was then watered with NaCl solution every 3 days. The initial NaCl concentration was 100 mM, and it was then increased in 50-mM increments until a final concentration of 300 mM was reached.

Regarding floating leaf disk assays, 0.8-cm diameter leaf disks (six disks per treatment) were prepared from WT and transgenic leaves at identical developmental stages. The disks were floated on 0 mM (i.e., H_2_O) and 300 mM NaCl solutions for 5 days, and they were then treated with 80% aqueous acetone, and the total chlorophyll content was calculated as previously described (Marr et al., 1995). The assays was repeated three times, and mean values were used for analyses.

### GLV Analysis

*Z*-3-hexenal, *E*-2-hexenal, and *Z*-3-hexenol were analyzed using a gas chromatography-mass spectrometry system coupled to a thermal desorption unit (TD-GC-MS). The TD-GC-MS analysis was completed using a GC-MS-QP 2010 Ultra instrument (Shimadzu Corporation, Japan) equipped with an Rtx-5MS column (30 m in length, 0.25 mm internal diameter, and 0.25 μm film thickness; Restek, United States) (Kallenbach et al., 2014). The generated data were processed using GC-MS Solution software (version 4.20, Shimadzu Corporation). *E*-2-hexenal and *Z*-3-hexenol were identified based on comparisons with pure standards, while *Z*-3-hexenal was identified by matching the mass spectrum with data in the NIST14 library and a previously reported retention time (Kallenbach et al., 2014). The peak area of each GLV was normalized based on the peak area at 15.5 min for PDMS tubing pieces, because this peak area was proportional to the PDMS tubing length.

### ADH Activity Measurements

Aliquots of leaf tissue extracts were stored at -80°C until assayed. ADH activity was determined colorimetrically (FLUOstar^®^ Omega) by quantifying the amount of NADH produced using an Alcohol Dehydrogenase Activity Colorimetric Assay Kit (Biovision).

### Volatile Treatment

Two-week-old *N. benthamiana* plants grown on Murashige and Skoog agar plates (250 cm^3^) were treated with dichloromethane (DCM; Sigma–Aldrich) or individual GLVs (i.e., *E*-2-hexenal, *Z*-3-hexenol, and Z-3-hexenyl acetate; Sigma–Aldrich). Volatiles were diluted with DCM, which does not induce *HPL* expression. A 2-μL aliquot of 0.1 M volatile solution was applied to 3M^TM^ Micropore^TM^ Surgical Tape, which was attached to the inside of the plate cover. The cover was immediately set on the plastic plate, and the plants were incubated for 1 h at 25°C in an illuminated growth chamber (70 μmol m^-2^ s^-1^). DCM-treated plants were used as controls.

### Statistical Analysis

All experiments were repeated three times, and mean values were analyzed with Student’s *t*-test implemented in the JMPIN program (version: 4.0.4).

## Results

### Sysr1 Amino Acid Sequence Exhibits Characteristics Typical of Medium-Chain ADHs

To identify components of the salt-tolerance pathway, we compared the gene expression levels of two strains of *Synechocystis* sp., namely hypersaline lake isolate PCC 6906 (Taxonomy ID 722431) and freshwater isolate PCC 6803 (Taxonomy ID 1148), in response to a high NaCl concentration (data not shown). We isolated salt-responsive gene 1 (designated *Synechocystis* salt responsive gene 1 (*sysr1*)) from PCC 6906. A BLAST search against the NCBI non-redundant protein sequence database with the Sysr1 amino acid sequence as the query revealed that Sysr1 is more than 80% identical to AdhA (slr1192 protein). The *Synechocystis* sp. strain PCC 6803 *slr1192* gene encodes a member of the medium-chain ADH family. Additionally, AdhA exhibits NADP-dependent ADH activity, with diverse primary alcohols and aldehydes as substrates (Vidal et al., 2009). An alignment of horse liver ADH (LADH, *Equus caballus*), AdhA (PCC6803), and Sysr1 (PCC6906) amino acid sequences is presented in **Supplementary Figure S1**. LADH has previously been used as a standard for comparisons of ADH structures (Eklund et al., 1976). Each ADH sample had the following two major domains: a substrate-binding or catalytic domain, consisting of an N-terminal region with irregular β-coils and a short C-terminal region; and a co-enzyme–binding domain, comprising a duplicated β-sheet known as a Rossmann fold (Rossmann et al., 1974) (**Supplementary Figure S1**). An analysis of the aligned sequences indicated that the Sysr1 amino acid sequence had characteristics typical of medium-chain ADHs.

### Sysr1 Functions as an ADH in *sysr1*-Transgenic *N. benthamiana* Plants

**FIGURE 1:**
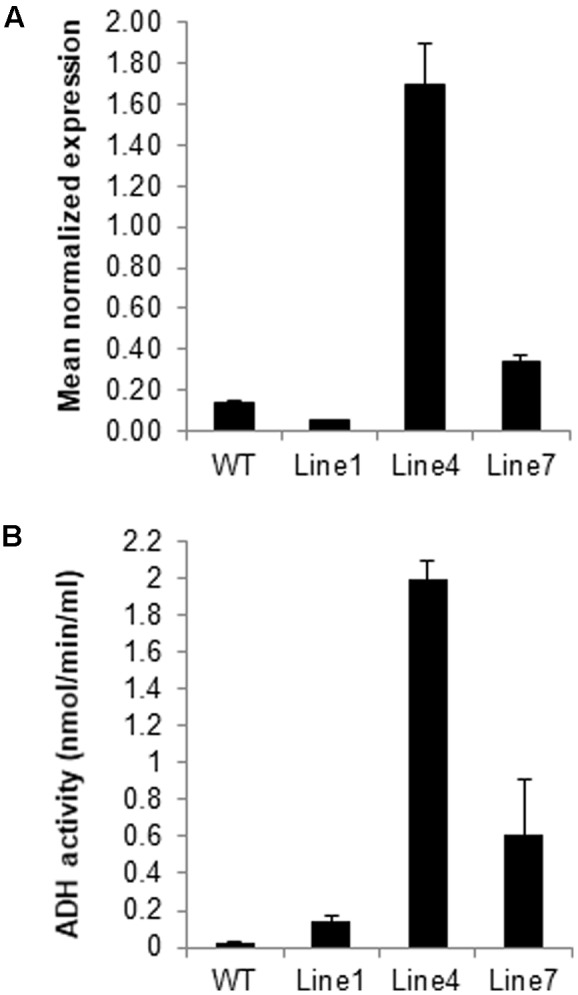
Identification of *sysr1*-transgenic plants. **(A)** Results of the qRT-PCR analyses of *sysr1* expression in three transgenic lines (Lines 1, 4, and 7) used for salt-tolerance assays. The *sysr1* transcription level was expressed as relative mRNA abundance (2^-ΔΔ*C*_t_^). Expression data were normalized against *Nicotiana benthamiana EF1*α transcript levels. For each sample, three biological replicates were analyzed with corresponding technical replicates. **(B)** Leaf ADH activity was compared among control and transgenic *N. benthamiana* plants. Control: WT plants. Lines 1, 4, and 7: homozygous transgenic lines (T_3_ generation).

The expression of *adhA* (*slr1192*) is induced by osmotic (Mikami et al., 2002), salt (Shoumskaya et al., 2005), and heat (Vidal et al., 2009) stresses. To investigate the potential role of Sysr1 in response to salinity stress, transgenic *N. benthamiana* plants that ectopically express *sysr1* were generated (**Supplementary Figure S2A**), and seven independently transformed tobacco lines were isolated (**Supplementary Figure S2B**). To assess *sysr1* expression levels in transgenic *N. benthamiana* plants, 3-week-old homozygous T_3_ transgenic seedlings were analyzed using qRT-PCR (**Figure 1A**). We then used an enzyme activity assay to confirm the mRNA data (**Figure 1B**). Lines 4 and 7 exhibited the highest *sysr1* expression levels and ADH activity, so the lines were selected for further analyses (**Figures 1A,B**). WT and Line 1 plants exhibited similarly low *sysr1* expression levels and ADH activity, so Line 1 was used as a control in subsequent experiments (**Figures 1A,B**). There were no obvious phenotypic differences between the transgenic and WT plants under normal growth conditions (**Supplementary Figure S2C**).

### Positive Correlation between ADH Activity and Salt Tolerance in *sysr1*-Transgenic Plants

**FIGURE 2:**
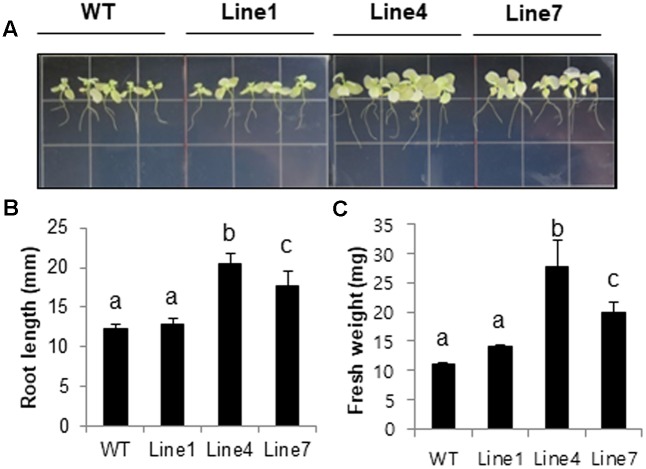
Effects of *sysr1* overexpression on salt tolerance during the seedling stage. **(A–C)** Phenotypes of WT and transgenic plants grown on Murashige and Skoog medium supplemented with 300 mM NaCl. Two-week-old WT and transgenic plants (Lines 1, 4, and 7) grown on Murashige and Skoog agar were transferred to the same medium containing 300 mM NaCl, and plants were allowed to grow for an additional 4 weeks. Plants were photographed at the conclusion of the salt treatment. **(B)** Analysis of root length and **(C)** whole-seedling fresh weight. The data for three biological replicates were averaged. Different letters represent significant differences at *P* < 0.05 (*t*-test). Bars indicate the standard deviation of the mean.

**FIGURE 3:**
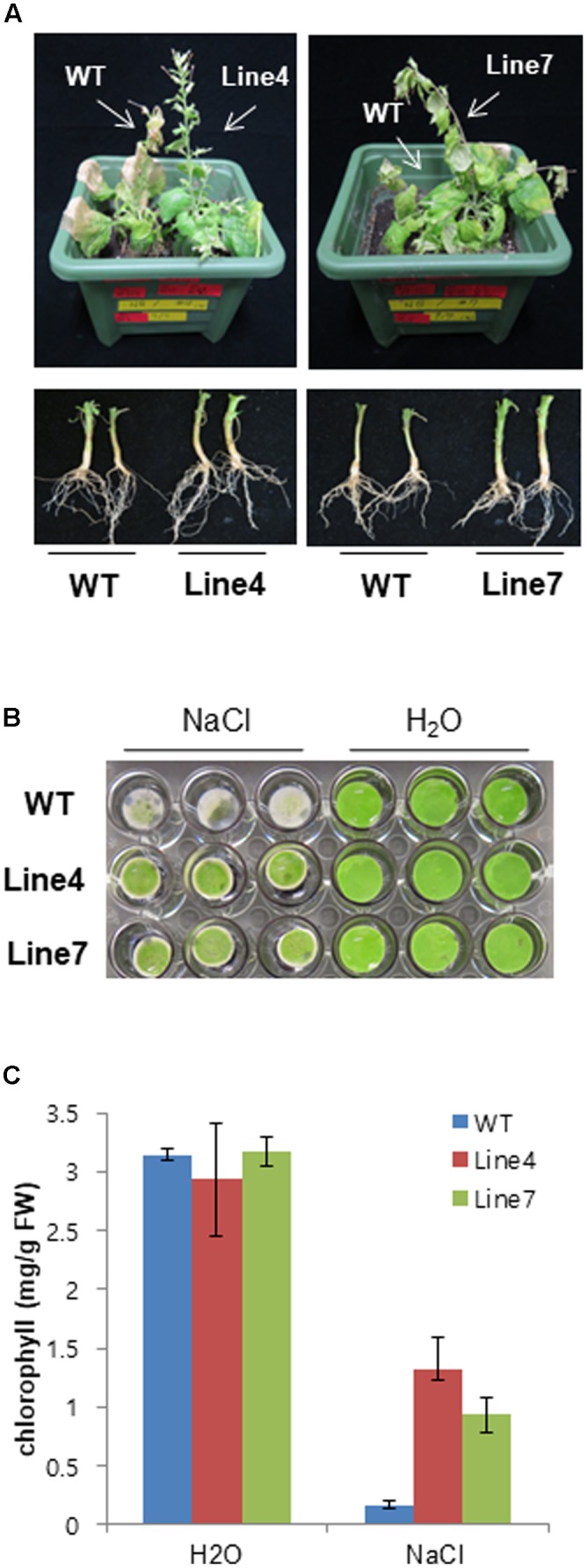
Effects of *sysr1* overexpression on salt tolerance during the adult stage. **(A)** Phenotype of WT and transgenic plants watered with NaCl solution (from 100 to 300 mM) for 4 weeks. **(B)** Representative image depicting phenotypic differences between WT and transgenic leaf disks. Leaves of 4-week-old WT and transgenic plants were cut into leaf disks (0.8 cm diameter) and floated on H_2_O or 300 mM NaCl. Leaf disks were incubated for 5 days at 25°C under a 16-h:8-h dark photoperiod. **(C)** Chlorophyll contents of leaf disks from WT and transgenic plants treated with H_2_O or 300 mM NaCl. Damages caused by salt stress are indicated by the extent of leaf-tissue bleaching after 5 days (*n* = 9).

Plant salt tolerance can be assessed based on the relative plant growth rate after prolonged exposure to a given salt concentration or the plant survival rate after a treatment with a defined salt concentration (Munns, 2002). We analyzed WT and transgenic plants to investigate whether the constitutive expression of *sysr1* enhances salt tolerance. Two-week-old seedlings were transferred to plates containing Murashige and Skoog agar medium supplemented with 300 mM NaCl. After 1 month, primary root length and fresh weight data were analyzed. The transgenic seedlings from Lines 4 and 7 grew better than the WT plants (**Figures 2A–C**). Furthermore, the 300 mM NaCl treatment considerably inhibited the growth of WT and Line 1 control plants, resulting in lower fresh weights than the seedlings of Lines 4 and 7 (**Figure 2B**). We also assessed plant growth in response to salinity in adult plants grown in soil. Six-week-old WT and *sysr1*-OX plants grown in the same pot were watered with NaCl solution (100–300 mM) for 1 month. As shown in **Figure 3A**, WT plants exhibited chlorosis and growth retardation, whereas *sysr1*-OX tobacco plants grew relatively well, thus demonstrating that ectopic expression of *sysr1* significantly enhanced the tolerance of these transgenic plants to salinity. The degree of leaf bleaching provides a visual estimate of the damage caused by salt stress. The effects of salinity stress on chlorophyll content were measured using a floating leaf disk assay. When leaf disks were floated on a 300 mM NaCl solution for 5 days, the disks of WT plants were bleached more intensely than those of *sysr1*-OX plants (**Figures 3B,C**). Additionally, decreases in leaf disk chlorophyll levels were greater in WT plants than in *sysr1*-OX plants (**Figures 3B,C**). These results indicated that transgenic *N. benthamiana* plants overexpressing *sysr1* were better able to tolerate salinity stress than WT plants. Plant damage caused by high salt concentrations likely varies depending on the age of the plant, and inhibited root growth was clearly observed during the seedling stage. However, in adults, inhibited growth of aerial plant parts and chlorosis of the leaves were more prominent symptoms of salt stress (**Figure 3A**). These results suggested a positive correlation between ADH activity and salt tolerance in *sysr1*-OX plants.

To investigate whether this salinity tolerance was attributable to osmotic mechanisms, 2-week-old WT and *sysr1*-OX seedlings were exposed to mannitol (400 mM). Osmotic stress tolerance was assessed by monitoring primary root elongation 4 weeks later. A significant difference in the root growth rate was observed between transgenic and WT seedlings (**Supplementary Figure S3**) and these results implied that *sysr1* overexpression conferred tolerance to salinity and osmotic stresses.

### *Z*-3-Hexenol Was More Abundant Than *Z*-3-Hexenal in Transgenic Tobacco Plants

**FIGURE 4:**
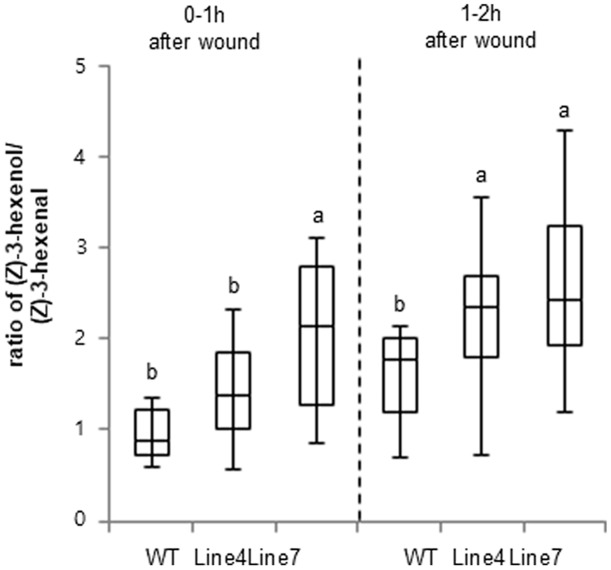
Changes in ADH activity influenced the quality of the emitted wound-induced GLVs. Leaves from WT and *sysr1*-transgenic plants were wounded (one leaf per plant), and were immediately placed in a ventilated PET cup containing two PDMS tubes. After 1–2 h, PDMS tubes were collected and analyzed. The peak areas of *Z*-3-hexenol and *Z*-3-hexenal (mean + standard deviation; *n* = 8) were determined as representative values. Different letters represent significant differences at *P* < 0.05 (*t*-test).

Previous studies indicated that ADH is responsible for the conversion of C_6_ aldehydes to their corresponding alcohols (Bicsak et al., 1982; Longhurst et al., 1990). Therefore, we compared the quality of the emitted GLVs between WT and *sysr1*-OX plants. The volatiles from transgenic leaves with enhanced ADH levels were analyzed using GC-MS (Kallenbach et al., 2014) to determine the effects of *sysr1* overexpression on the relative amount of volatile aldehydes and alcohols in wounded leaves. The results indicated that Sysr1 was involved in the interconversion of aldehydes and alcohols in transgenic *N. benthamiana* leaves. The WT control plants produced more *Z*-3-hexenal than *Z*-3-hexenol within 1 h of wounding (**Figure 4**). However, leaf *Z*-3-hexenol levels were approximately twofold higher in *sysr1*-OX plants than in WT plants within 1 h of wounding. Results of the GC-MS analysis revealed that the conversion of *Z*-3-hexenal (C_6_ aldehyde) to its corresponding alcohol, *Z*-3-hexenol (C_6_ alcohol), was at least partially mediated by Sysr1 in transgenic leaves. These data indicated that Sysr1 functions as an ADH in *sysr1*-OX plants.

### Expression of Stress-Related Genes Is Altered in *sysr1*-OX *N. benthamiana* Plants

**FIGURE 5:**
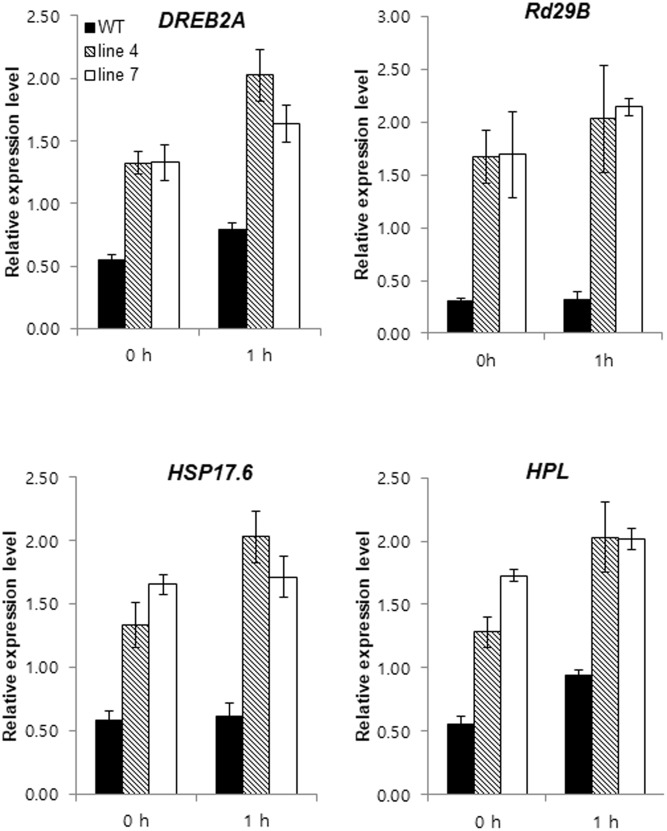
Expression patterns of genes with known roles in abiotic stress responses in tobacco plants. Gene expression levels in WT and *sysr1-*transgenic leaves (Lines 4 and 7) were compared by qRT-PCR after 0 and 1 h of treatment with 300 mM NaCl. Error bars indicate the standard deviation of three technical replicates, and the results were consistent in two biological replicates. *EF1*α was used as a reference gene. *HPL*, hydroperoxide lyase; *DREB2A*, dehydration-responsive element binding protein 2a; *RD29B*, responsive to desiccation 29*; HSP17.6*, heat shock protein 17.6.

The expression of some stress-related genes was analyzed using qRT-PCR to determine how *sysr1* overexpression increased salt tolerance. Genes encoding key signaling factors for abiotic stress response pathways [e.g., dehydration-responsive element binding protein 2a (*DREB2A*), heat shock protein 17.6 (*HSP17.6*), responsive to desiccation 29 (*RD29B*), and *HPL*] were more highly expressed in transgenic plants than in WT plants under normal growth conditions and after treatment with 300 mM NaCl (**Figure 5**). We sampled plants 1 h after salt treatment to highlight the phenotypes of *sysr1*-OX plants. Even in the absence of salt stress, greater accumulation of transcripts associated with stress-related genes was observed in *sysr1*-transgenic plants compared to WT plants. This phenomenon is similar to the priming effect observed in plants exposed to salt stress in advance. Therefore, these observations implied that the greater salt tolerance of *sysr1*-OX plants was relevant to the elevated expression levels of stress response genes.

### Airborne Signals from Salt-Stressed *sysr1*-OX Plants Induce Salt Tolerance in Neighboring WT Seedlings

**FIGURE 6:**
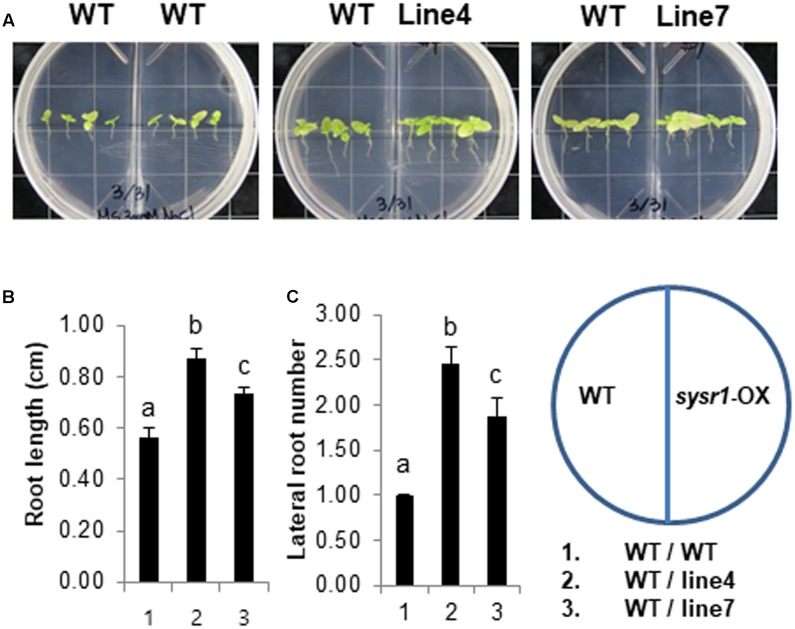
Effects of VOCs emitted from salt-stressed *sysr1*-OX plants on the salt tolerance of neighboring WT plants. **(A–C)** Two-week-old WT and transgenic plants (Lines 4 and 7) grown on Murashige and Skoog agar medium were transferred to the same medium containing 300 mM NaCl, and plants were allowed to grow for an additional 4 weeks. We used two-compartment plates with WT seedlings in one compartment and *sysr1*-OX or WT seedlings in the other. **(A)** Phenotypes of WT and transgenic plants grown on Murashige and Skoog agar medium supplemented with 300 mM NaCl. **(B)** Root length analysis of WT plants grown with *sysr1*-OX plants. **(C)** Analysis of lateral root numbers in WT plants grown with *sysr1*-OX plants. All values are mean ± SD of three independent experiments (*n* = 8 seedlings per experiment). Different letters represent significant differences at *P* < 0.05 (*t*-test). Bars indicate the standard deviation of the mean.

Environmental stresses increase the quantity and quality of volatile organic compounds (VOCs) emitted by plants (Loreto and Schnitzler, 2010). A recent study concluded that salt-responsive *A. thaliana* VOCs induce salt tolerance in neighboring plants (Lee and Seo, 2014). To determine whether salt stress promotes the emission of VOCs from *sysr1*-OX plants to enhance salt tolerance in neighboring WT plants, we treated WT and *sysr1*-OX plants with 300 mM NaCl and investigated whether VOCs released from the *sysr1-*transgenic plants induced salt tolerance in WT plants. Two-week-old WT and *sysr1-*OX seedlings were transferred to MS agar medium supplemented with 300 mM NaCl. We used two-compartment plates, which contained WT seedlings in one compartment and *sysr1*-OX or WT seedlings in the other. The plates, which allowed the exchange of airborne signals between compartments, were sealed and incubated at 25 ± 1°C for 4 weeks. We then examined the growth of WT plants that were grown with WT or *sysr1*-transgenic plants exposed to 300 mM NaCl. The salt tolerance of WT plants was enhanced in the presence of *sysr1*-transgenic plants, suggesting that the VOCs emitted from the *sysr1*-transgenic plants enhanced the salt tolerance of neighboring WT plants (**Figures 6A–C**). We also assessed the volatile effects of *sysr1*-OX plants on salt tolerance in adult plants grown in soil. WT and *sysr1*-OX plants grown in the same pot received water supplemented with NaCl (100–300 mM). Unfortunately, we did not observe acquired salt tolerance in WT plants that were grown together with adult *sysr1*-OX plants in the same pot (**Figure 3A**).

### Green Leaf Volatiles Strongly and Rapidly Induce Stress-Related Gene Expression

**FIGURE 7:**
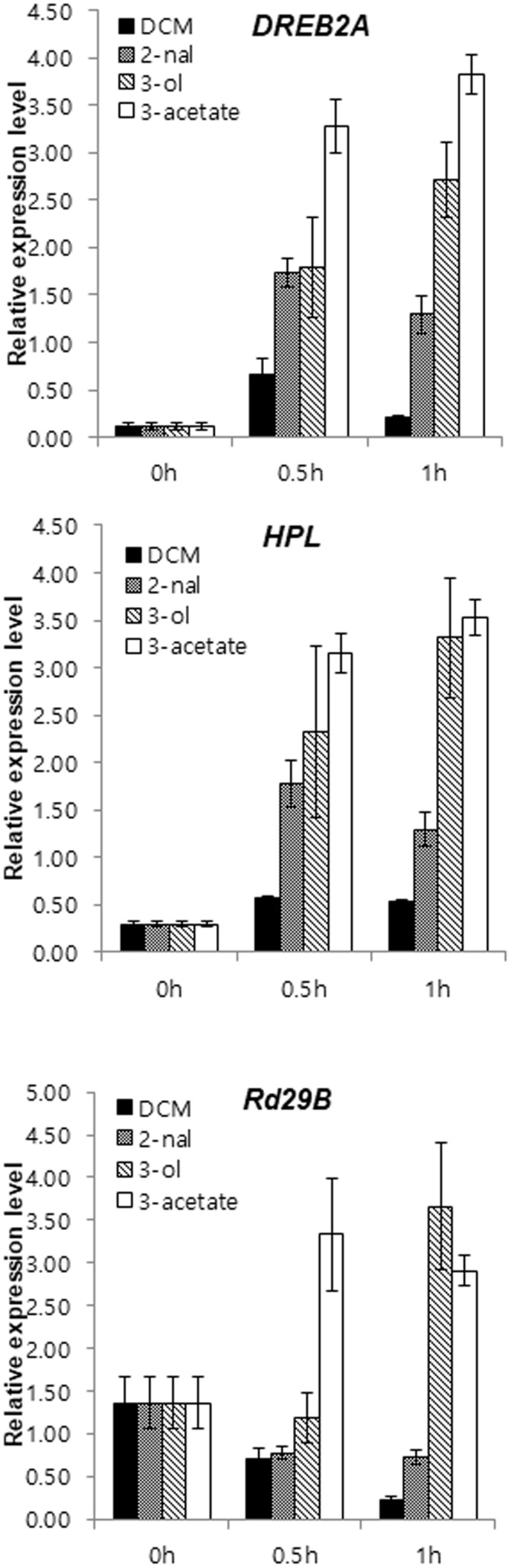
Expression levels of abiotic stress-related genes in *N. benthamiana* were upregulated in response to GLV treatments. Gene expression is presented as relative transcript abundance of *N. benthamiana* leaves (WT) treated with 2 μL 0.1 M DCM, *E*-2-hexenal, *Z*-3-hexenol, or *E*-3-hexenyl acetate for 30 and 60 min. Error bars indicate the standard deviation of three technical replicates, and the results were consistent in two biological replicates. The *EF1*α gene was used as a reference gene. *HPL*, hydroperoxide lyase; *DREB2A*, dehydration-responsive element binding protein 2a; *RD29B*, responsive to desiccation 29.

Even in the absence of salt stress, *HPL* transcripts accumulated more in *sysr1*-transgenic plants than in WT plants (**Figure 5**). *HPL* is important for GLV biosynthesis in *N. attenuata* (Allmann et al., 2010). Additionally, *sysr1* overexpression modifies the balance between *Z*-3-hexenal and *Z*-3-hexenol in transgenic leaves. Therefore, we speculated that GLVs might be airborne signals. Furthermore, *sysr1*-OX and WT plants may differ with regard to the quality or quantity of GLVs emitted in response to high-salt conditions. To elucidate the molecular mechanisms underlying the induction of salt tolerance in plants neighboring *sysr1*-OX seedlings, we compared the effects of GLVs on the expression of defense-related genes. GLVs comprise a family of C_6_ compounds, including *E*-2-hexenal, *Z*-3-hexenol, and hexenyl derivative *Z*-3-hexenyl acetate. WT plants were treated with pure vaporized C_6_ compounds (10 nmol cm^-3^ for 1 h). The seedlings were collected 0.5 and 1 h after initiating treatment, because the focus of this study was early transcriptional changes induced by GLVs. Earlier studies revealed that *DREB2A* transcript levels were highest 0.5 h after samples were exposed to *E*-2-hexenal (Yamauchi et al., 2015). We observed a transient increase in *DREB2A* transcript abundance at 0.5 h, and a subsequent decrease was detected after 1 h in WT plants treated with vaporized *E*-2-hexanal. In contrast, *DREB2A* transcription levels in DCM-treated control plants remained low (**Figure 7**). We also tested the effects of other GLVs on selected stress-related transcript, and vaporized *Z*-3-hexenol and *Z*-3-hexenyl acetate induced the expression of *DREB2A*, *RD29B*, and *HPL* (**Figure 7**). Moreover, *Z*-3-hexenol and *Z*-3-hexenyl acetate upregulated the expression of selected stress-related genes more than *E*-2-hexenal (**Figure 7**).

### Treatments with Pure C_6_ Compounds Can Induce Salt Tolerance

**FIGURE 8:**
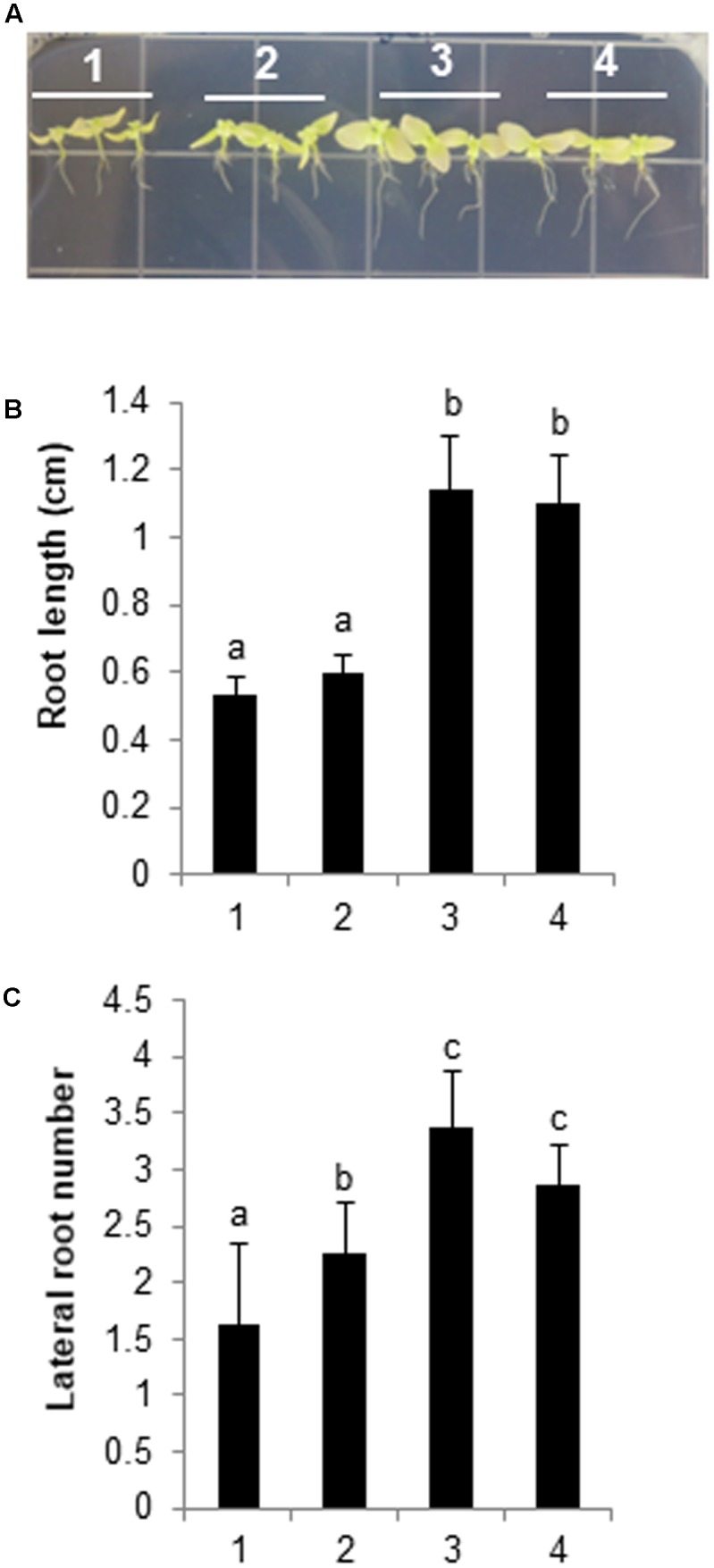
Biological effects of GLV treatments on *N. benthamiana* plants. Two-week-old seedlings were pretreated with 2 μl 0.1 M GLV or DCM (control) for 1 h. The seedlings were then transferred to Murashige and Skoog agar medium containing 300 mM NaCl, and plants were allowed to grow for an additional 4 weeks. The plants were photographed at the conclusion of the salt treatment. **(A)** (1) DCM; (2) *E*-2-hexenal; (3) *Z*-3-hexenol; (4) *Z*-3-hexenyl acetate. **(B)** Analysis of root length and **(C)** number of lateral roots. The data for three biological replicates were averaged. Different letters represent significant differences at *P* < 0.05 (*t*-test). Bars indicate the standard deviation of the mean.

We assessed GLV-induced salt tolerance based on root length and the number of lateral roots, because GLV treatments enhanced the expression of *HPL*, *DREB2A*, and *RD29B* (**Figure 7**), which contribute to abiotic stress tolerance (Sakuma et al., 2006a). After 2 weeks of growth on basal Murashige and Skoog agar plates, WT seedlings were treated with DCM (i.e., vaporized solvent control) and three synthetic GLVs for 1 h, and samples were then transferred to vertical Murashige and Skoog agar plates supplemented with 300 mM NaCl. Regarding primary root length and the number of lateral roots, seedlings pretreated with *Z*-3-hexenol or *Z*-3-hexenyl acetate grew better than solvent control-treated seedlings, thus indicating the physiological importance of GLVs in salt-stress responses. However, the *E*-2-hexenal pretreatment did not enhance salt tolerance (**Figure 8**), and these results were consistent with the expression patterns of stress-related genes in GLV-treated seedlings (**Figure 7**). Comparing salt-induced growth inhibition after treatments with three different GLVs indicated that C_6_ alcohol and C_6_ ester forms of GLVs increased salt tolerance more than the C_6_ aldehyde form.

## Discussion

Soil salinity is a major abiotic stress that adversely affects plant growth and productivity. Excessive salt entering plant cells can trigger ionic imbalances that cause respiratory and photosynthetic complications, which ultimately lead to inhibited growth, injury, and even death in severe cases (Munns, 2002). In this study, we revealed that Sysr1 helps regulate plant salt-stress tolerance. The ectopic expression of *sysr1* in transgenic *N. benthamiana* plants enhanced salt tolerance. The encoded ADH enhanced the conversion of aldehydes to alcohols, thus affecting the balance between C_6_ aldehydes and alcohols in *sysr1-*transgenic plants. Furthermore, airborne signals from salt-stressed *sysr1*-OX plants triggered salinity tolerance in neighboring WT plants. We hypothesized that communication between *sysr1*-transgenic and WT plants under high-salt conditions helps WT plants cope with subsequent exposures to salt stress. To test this hypothesis, we investigated the effects of GLV treatments on salt-stress tolerance. The *Z*-3-hexenol and *Z*-3-hexenyl acetate treatments upregulated *HPL, DREB2A*, and *RD29B* expression levels, and substances also alleviated the growth inhibition of WT plants exposed to salinity stress. These results suggested that Sysr1 affects the quality of GLV components, resulting in physiological implications for salt tolerance.

### How Does *sysr1* Improve the Salt Tolerance of Transgenic Plants?

Besides well-established ADH functions that occur during seed development and responses to flooding stress, there is mounting evidence that ADH mediates tolerance to other abiotic stresses. Several previous studies of model plants confirmed that *ADH* expression is influenced by stress (Matton et al., 1990; Christie et al., 1991; Ingersoll et al., 1994; Bucher et al., 1995), and it is also linked to changes in secondary metabolism (Bicsak et al., 1982; Longhurst et al., 1990; Speirs et al., 1998). Although *ADH* expression is induced by salt stress (Vidal et al., 2009; Zhang et al., 2016), the role of ADH during salt-tolerance signaling has not been established. In this study, we tested whether transgenic plants carrying *Synechocystis* sp. *ADH* exhibit salinity tolerance. We also attempted to characterize the mechanism responsible for the correlation between salinity tolerance and ectopic *sysr1* expression (**Figures 2**, **3**). Our findings clearly indicated that *sysr1* overexpression increases the salt tolerance of *N. benthamiana* plants.

The results of our gene expression analyses may help explain increased salt tolerance of *sysr1*-OX plants. The overexpression of *sysr1* in *N. benthamiana* plants upregulated the expression of the abiotic stress-related genes *DREB2A*, *RD29B*, *HSP17.6*, and *HPL* (**Figure 5**). The enhanced expression of *DREB2A* reportedly increases rice tolerance to dehydration and salt stress conditions (Mallikarjuna et al., 2011), and *RD29A* and *RD29B* are specific targets of the DREB2A transcription factor (Sakuma et al., 2006a,b). These homologous genes are highly sensitive to various abiotic stressors. For example, cold, drought, and salt stresses induce *RD29A* and *RD29B* expression. However, the *RD29A* promoter is more responsive to drought and cold stresses, and the *RD29B* promoter is highly responsive to salt stress (Msanne et al., 2011). *HSP17.6A* encodes a small heat-shock protein belonging to the *A. thaliana* cytosolic class II family, and it is expressed during development and stress responses. Furthermore, overproduction of *HSP17.6A* and *NtHSP70-1* can increase salt or drought tolerance in plants (Sun et al., 2001; Cho and Hong, 2006). Because the expression levels of abiotic stress-related genes increased in *sysr1*-OX plants, we speculated that stress-induced signal transduction occurs faster in transgenic plant cells, resulting in faster and stronger activation of salt-tolerance-related responses. Upon exposure to salt stress, a set of signaling proteins is activated, thus augmenting salt-stress responses.

### How Does Sysr1 Affect the Transcription of Abiotic Stress-Related Genes, Including *DREB2A*, in Transgenic *N. benthamiana*?

A distinct characteristic of *sysr1*-transgenic plants is the exhibition of greater ADH activity than the WT plants. Consequently, *Z*-3-hexenol was more abundant than *Z*-3-hexenal (**Figure 4**). Previous reports indicated that *adh1* mutant plants released less hexanol and *Z*-3-hexenol than WT plants, but more *E*-2-hexenal was produced (Chang and Meyerowitz, 1986; Strommer, 2011). The overexpression of *ADH* in tomato plants changes the balance between the C_6_ aldehydes and alcohols in ripened fruits (Speirs et al., 1998). We speculated that the increase in *Z*-3-hexenol content in *sysr1*-OX *N. benthamiana* plants may influence the transcription of abiotic stress-related genes. Short-chain leaf volatiles (e.g., *E*-2-hexenal) can strongly induce the expression of abiotic stress-related transcription factor genes such as *DREB2A* (Yamauchi et al., 2015). However, we observed that the expression levels of abiotic stress-related genes were more than twofold higher in plants treated with vaporized *Z*-3-hexenol and *Z*-3-hexenyl acetate than in plants exposed to *E*-2-hexenal (**Figure 7**). Thus, our data indicate that GLVs formed in *sysr1*-transgenic plants can upregulate gene expression, leading to stronger effects of *Z*-3-hexenol than *E*-2-hexenal.

### How Do *sysr1*-OX Plants Trigger Salinity Tolerance in Neighboring Plants?

Green leaf volatiles are produced in reactions catalyzed by HPL, which is a component of the lipoxygenase pathway. In the lipoxygenase/HPL pathway, the plant first produces C_6_ aldehydes, which are then converted to C_6_ alcohols (e.g., *Z*-3-hexenol) by ADH (Matsui, 2006). In plant communities, GLVs are important infochemicals that mediate plant–plant and plant–insect interactions. In particular, *Z*-3-hexenol and *Z*-3-hexenyl acetate are associated with plant–plant communication (Engelberth et al., 2004; Farag et al., 2005; Heil and Silva Bueno, 2007; Frost et al., 2008; Heil et al., 2008). Airborne *Z*-3-hexenol from wounded plants may trigger pre-defense reactions in neighboring healthy plants, enabling faster and stronger responses during subsequent attacks. This phenomenon is called plant–plant communication or the priming effect of volatiles (Wei and Kang, 2011), but the physiological and molecular mechanisms responsible for GLV-induced priming have not been characterized. Priming often results in the enhanced transcription of defense-related genes (Thulke and Conrath, 1998; van Wees et al., 1999; Zimmerli et al., 2000; Kohler et al., 2002). Thus, transcription factors are important for the regulation of priming effect initiation (Van der Ent et al., 2009).

Unfortunately, we were unable to identify the salt-induced GLVs released in WT and *sysr1*-transgenic plants, and this likely occurred because GLVs were released at very low levels. However, the results of our experiment using two-compartment plates suggested that airborne signals from salt-stressed *sysr1*-transgenic plants enhanced the salt tolerance of neighboring plants (**Figures 6A–C**). The priming effect was observed because plants were located in a small enclosed space, and neighboring plants were exposed to relatively high concentrations of volatile components for an extended period. In contrast, WT and *sysr1*-OX plants grown in soil (in an open space) did not exhibit a priming effect, because they were only briefly exposed to relatively low concentrations of volatile components. Therefore, exposure to sufficient concentrations of volatile components for an adequate period is required for the induction of a priming effect between neighboring plants. However, we observed that vaporized *Z*-3-hexenol and *Z*-3-hexenyl acetate considerably increased salt tolerance in neighboring WT plants (**Figure 8**). Interestingly, *E*-2-hexenal had relatively little priming effects. Shiojiri et al. (2012) exposed healthy *A. thaliana* plants to 140 ppt GLVs from wounded neighboring plants twice per week for 3 weeks, and this concentration triggered a response in the healthy plants. Although we were unable to measure salt-induced GLVs, the aforementioned results suggest that a very low concentration of salt-induced GLVs can trigger salt tolerance in neighboring plants. Therefore, increases in ADH activity may affect the salt tolerance of neighboring plants by changing the balance between emitted *Z*-3-hexenol and *E*-2-hexenal.

### A Proposed Role for Sysr1

In plants, ADH enzymes have multiple functions related to anaerobic and aerobic fermentation as well as the production of scents that discourage predation, attract pollinators, and facilitate seed dispersal. In particular, *sysr1* overexpression affects the quality of stress-inducible GLVs, resulting in the upregulation of expression of stress-related genes. These changes may be associated with observed enhanced salt tolerance of *sysr1*-OX plants and neighboring plants. Our results suggested that the increased salt tolerance of *sysr1*-OX plants may have resulted from increased expression of stress-related genes, which was caused by enhanced *Z*-3-hexenol production. In this study, we could not explain why the priming effect associated with the induction of salt tolerance in neighboring plants was only observed in seedlings cultivated in an airtight container. Experiments designed to fully characterize the molecular mechanisms associated with the regulation of the salt tolerance priming effect of *sysr1*-OX plants are currently in progress.

## Author Contributions

SY and SM designed the study. SY, SSK, H-JS, and S-KK conducted the experiments and analyzed the data. JP, JIL, ES, SC, JK, and MA collected plant materials. SWK, HP, WJ, YL, and JRL commented on the research. SY wrote the manuscript.

## Conflict of Interest Statement

The authors declare that the research was conducted in the absence of any commercial or financial relationships that could be construed as a potential conflict of interest. The reviewer HC declared a shared affiliation, with no collaboration, with several of the authors to the handling Editor.

## Footnotes

1 www.bio-rad.com

2 www.takara.co.kr

## Supplementary Material

**FIGURE S1 |** Alignment of *Synechocystis* sp. PCC 6906 (Sysr1), *Synechocystis* sp. PCC 6803 (AdhA), and horse liver (LADH) ADH sequences. Residues shared by *Synechocystis* sp. proteins are presented in gray, while amino acids conserved among all three ADHs are in black. The major functional domains are indicated, along with the binding sites of the catalytic (^∗^) and structural (•) Zn ions. The β sheets of the NAD-binding Rossmann fold are underlined. GenBank accessions for PCC 6906, PCC 6803, and horse liver ADHs are KY014075, WP_010874320, and NP_001075414.1, respectively.

**FIGURE S2 |** Analysis of *sysr1*-overexpressing transgenic *N. benthamiana* lines. **(A)** transformation of *N. benthamiana* plants. **(B)** Quantitative real-time PCR analyses of *sysr1* gene expression in WT plants and *sysr1*-overexpressing transgenic lines (T_1_). Total RNA was isolated from untreated plants and cDNA was synthesized as described in the “Materials and Methods” section. The *EF1*α gene was used as a reference gene. **(C)** Images of representative *sysr1* transformants and WT plants at 50 days.

**FIGURE S3 |** Effects of osmotic stress (400 mM mannitol) on shoot and root growth in the control and three transgenic plants (Lines 1, 4, and 7). **(A)** Plants were photographed after 4 weeks of stress treatment. Two-week-old WT and transgenic plants (Lines 1, 4, and 7), grown on Murashige and Skoog agar medium, were transferred to the same medium supplemented with 400 mM mannitol, and plants were allowed to grow for an additional 4 weeks. **(B)** Primary root lengths of WT and transgenic plants treated with 400 mM mannitol for 4 weeks (*n* = 12). The data for three biological replicates were averaged. Different letters represent significant differences at *P* < 0.05 (*t*-test). Bars indicate the standard deviation of the mean.

## References

[B1] Abebe T. Guenzi A. C. Martin B. Cushman J. C. (2003). Tolerance of mannitol-accumulating transgenic wheat to water stress and salinity. *Plant Physiol.* 131 1748–1755. 10.1104/pp.102.003616 12692333 PMC166930

[B2] Alka K. Windle H. J. Cornally D. Ryan B. J. Henehan G. T. M. (2013). A short chain NAD(H)-dependent alcohol dehydrogenase (HpSCADH) from *Helicobacter pylori*: a role in growth under neutral and acidic conditions. *Int. J. Biochem. Cell Biol.* 45 1347–1355. 10.1016/j.biocel.2013.04.006 23583739

[B3] Allmann S. Halitschke R. Schuurink R. C. Baldwin I. T. (2010). Oxylipin channelling in *Nicotiana attenuata*: lipoxygenase 2 supplies substrates for green leaf volatile production. *Plant Cell Environ.* 33 2028–2040. 10.1111/j.1365-3040.2010.02203.x 20584148

[B4] Bailey-Serres J. Voesenek L. A. (2008). Flooding stress: acclimations and genetic diversity. *Annu. Rev. Plant Biol.* 59 313–339. 10.1146/annurev.arplant.59.032607.092752 18444902

[B5] Bate N. J. Rothstein S. J. (1998). C6-volatiles derived from the lipoxygenase pathway induce a subset of defense-related genes. *Plant J.* 16 561–569. 10.1046/j.1365-313x.1998.00324.x 10036774

[B6] Behnke K. Kleist E. Uerlings R. Wildt J. Rennenberg H. Schnitzler J. P. (2009). RNAi-mediated suppression of isoprene biosynthesis in hybrid poplar impacts ozone tolerance. *Tree Physiol.* 29 725–736. 10.1093/treephys/tpp009 19324699

[B7] Bicsak T. A. Kann L. R. Reiter A. Chase T. Jr. (1982). Tomato alcohol dehydrogenase: purification and substrate specificity. *Arch. Biochem. Biophys.* 216 605–615. 10.1016/0003-9861(82)90250-8 7051979

[B8] Brilli F. Ruuskanen T. M. Schnitzhofer R. Muller M. Breitenlechner M. Bittner V. (2011). Detection of plant volatiles after leaf wounding and darkening by proton transfer reaction “time-of-flight” mass spectrometry (PTR-TOF). *PLOS ONE* 6:e20419. 10.1371/journal.pone.0020419 21637822 PMC3102719

[B9] Bucher M. Brander K. A. Sbicego S. Mandel T. Kuhlemeier C. (1995). Aerobic fermentation in tobacco pollen. *Plant Mol. Biol.* 28 739–750. 10.1007/BF00021197 7647304

[B10] Chang C. Meyerowitz E. M. (1986). Molecular cloning and DNA sequence of the *Arabidopsis thaliana* alcohol dehydrogenase gene. *Proc. Natl. Acad. Sci. U.S.A.* 83 1408–1412. 10.1073/pnas.83.5.1408 2937058 PMC323085

[B11] Chase T. (1999). Alcohol dehydrogenases: identification and names for gene families. *Plant Mol. Biol. Rep.* 17 333–350. 10.1023/A:1007620627083

[B12] Chattopadhyay A. Subba P. Pandey A. Bhushan D. Kumar R. Datta A. (2011). Analysis of the grasspea proteome and identification of stress-responsive proteins upon exposure to high salinity, low temperature, and abscisic acid treatment. *Phytochemistry* 72 1293–1307. 10.1016/j.phytochem.2011.01.024 21353267

[B13] Cho E. K. Hong C. B. (2006). Over-expression of tobacco NtHSP70-1 contributes to drought-stress tolerance in plants. *Plant Cell Rep.* 25 349–358. 10.1007/s00299-005-0093-2 16365678

[B14] Christie P. J. Hahn M. Walbot V. (1991). Low-temperature accumulation of alcohol dehydrogenase-1 mRNA and protein activity in maize and rice seedlings. *Plant Physiol.* 95 699–706. 10.1104/pp.95.3.699 16668042 PMC1077594

[B15] Conley T. R. Peng H. P. Shih M. C. (1999). Mutations affecting induction of glycolytic and fermentative genes during germination and environmental stresses in Arabidopsis. *Plant Physiol.* 119 599–608. 10.1104/pp.119.2.599 9952456 PMC32137

[B16] Conrath U. (2009). Priming of induced plant defense responses. *Plant Innate Immun.* 51 361–395.

[B17] Croft K. Juttner F. Slusarenko A. J. (1993). Volatile products of the lipoxygenase pathway evolved from *Phaseolus vulgaris* (L.) leaves inoculated with *Pseudomonas syringae* pv *phaseolicola*. *Plant Physiol.* 101 13–24. 10.1104/pp.101.1.13 12231661 PMC158642

[B18] D’Auria J. C. Pichersky E. Schaub A. Hansel A. Gershenzon J. (2007). Characterization of a BAHD acyltransferase responsible for producing the green leaf volatile (Z)-3-hexen-1-yl acetate in Arabidopsis thaliana. *Plant J.* 49 194–207. 10.1111/j.1365-313X.2006.02946.x 17163881

[B19] de Bruxelles G. L. Peacock W. J. Dennis E. S. Dolferus R. (1996). Abscisic acid induces the alcohol dehydrogenase gene in Arabidopsis. *Plant Physiol.* 111 381–391. 10.1104/pp.111.2.381 8787023 PMC157847

[B20] Dolferus R. Debruxelles G. Dennis E. S. Peacock W. J. (1994). Regulation of the *Arabidopsis Adh*-gene by anaerobic and other environmental stresses. *Ann. Bot.* 74 301–308. 10.1006/anbo.1994.1121

[B21] Eklund H. Nordstrom B. Zeppezauer E. Soderlund G. Ohlsson I. Boiwe T. (1976). Three-dimensional structure of horse liver alcohol dehydrogenase at 2-4 A resolution. *J. Mol. Biol.* 102 27–59. 10.1016/0022-2836(76)90072-3 178875

[B22] Engelberth J. Alborn H. T. Schmelz E. A. Tumlinson J. H. (2004). Airborne signals prime plants against insect herbivore attack. *Proc. Natl. Acad. Sci. U.S.A.* 101 1781–1785. 10.1073/pnas.0308037100 14749516 PMC341853

[B23] Farag M. A. Fokar M. Zhang H. A. Allen R. D. Pare P. W. (2005). (Z)-3-Hexenol induces defense genes and downstream metabolites in maize. *Planta* 220 900–909. 10.1007/s00425-004-1404-5 15599762

[B24] Frost C. J. Appel M. Carlson J. E. De Moraes C. M. Mescher M. C. Schultz J. C. (2007). Within-plant signalling via volatiles overcomes vascular constraints on systemic signalling and primes responses against herbivores. *Ecol. Lett.* 10 490–498. 10.1111/j.1461-0248.2007.01043.x 17498148

[B25] Frost C. J. Mescher M. C. Dervinis C. Davis J. M. Carlson J. E. De Moraes C. M. (2008). Priming defense genes and metabolites in hybrid poplar by the green leaf volatile cis-3-hexenyl acetate. *New Phytol.* 180 722–733. 10.1111/j.1469-8137.2008.02599.x 18721163

[B26] Gomi K. Yamasaki Y. Yamamoto H. Akimitsu K. (2003). Characterization of a hydroperoxide lyase gene and effect of C6-volatiles on expression of genes of the oxylipin metabolism in Citrus. *J. Plant Physiol.* 160 1219–1231. 10.1078/0176-1617-01177 14610891

[B27] Hanson A. D. Jacobsen J. V. Zwar J. A. (1984). Regulated expression of three alcohol dehydrogenase genes in barley aleurone layers. *Plant Physiol.* 75 573–581. 10.1104/pp.75.3.573 16663668 PMC1066957

[B28] Heil M. Lion U. Boland W. (2008). Defense-inducing volatiles: In search of the active motif. *J. Chem. Ecol.* 34 601–604. 10.1007/s10886-008-9464-9 18408973 PMC2373414

[B29] Heil M. Silva Bueno J. C. (2007). Within-plant signaling by volatiles leads to induction and priming of an indirect plant defense in nature. *Proc. Natl. Acad. Sci. U.S.A.* 104 5467–5472. 10.1073/pnas.0610266104 17360371 PMC1838500

[B30] Hoog J. O. Stromberg P. Hedberg J. J. Griffiths W. J. (2003). The mammalian alcohol dehydrogenases interact in several metabolic pathways. *Chem. Biol. Interact.* 14 175–181. 10.1016/S0009-2797(02)00225-9 12604202

[B31] Ingersoll J. C. Rothenberg M. Liedl B. E. Folkerts K. Garvin D. Hanson M. R. (1994). A novel anther-expressed *adh*-homologous gene in *Lycopersicon esculentum*. *Plant Mol. Biol.* 26 1875–1891. 10.1007/BF00019500 7858224

[B32] Jornvall H. Hedlund J. Bergman T. Kallberg Y. Cederlund E. Persson B. (2013). Origin and evolution of medium chain alcohol dehydrogenases. *Chem. Biol. Interact.* 202 91–96. 10.1016/j.cbi.2012.11.008 23200944

[B33] Jornvall H. Hedlund J. Bergman T. Oppermann U. Persson B. (2010). Superfamilies SDR and MDR: from early ancestry to present forms. Emergence of three lines, a Zn-metalloenzyme, and distinct variabilities. *Biochem. Biophys. Res. Commun.* 396 125–130. 10.1016/j.bbrc.2010.03.094 20494124

[B34] Kallenbach M. Oh Y. Eilers E. J. Veit D. Baldwin I. T. Schuman M. C. (2014). A robust, simple, high-throughput technique for time-resolved plant volatile analysis in field experiments. *Plant J.* 78 1060–1072. 10.1111/tpj.12523 24684685 PMC4190661

[B35] Kasuga M. Liu Q. Miura S. Yamaguchi-Shinozaki K. Shinozaki K. (1999). Improving plant drought, salt, and freezing tolerance by gene transfer of a single stress-inducible transcription factor. *Nat. Biotechnol.* 17 287–291. 10.1038/7036 10096298

[B36] Kato-Noguchi H. (2001). Wounding stress induces alcohol dehydrogenase in maize and lettuce seedlings. *Plant Growth Regul.* 35 285–288. 10.1023/A:1014489922792

[B37] Kennedy R. A. Rumpho M. E. Fox T. C. (1992). Anaerobic metabolism in plants. *Plant Physiol.* 100 1–6. 10.1104/pp.100.1.1 16652929 PMC1075508

[B38] Kohler A. Schwindling S. Conrath U. (2002). Benzothiadiazole-induced priming for potentiated responses to pathogen infection, wounding, and infiltration of water into leaves requires the NPR1/NIM1 gene in Arabidopsis. *Plant Physiol.* 128 1046–1056. 10.1104/pp.010744 11891259 PMC152216

[B39] Lee K. Seo P. J. (2014). Airborne signals from salt-stressed *Arabidopsis* plants trigger salinity tolerance in neighboring plants. *Plant Signal. Behav.* 9:e28392. 10.4161/psb.28392 24603614 PMC4091499

[B40] Longhurst T. J. Tung H. F. Brady C. J. (1990). Developmental regulation of the expression of alcohol-dehydrogenase in ripening tomato fruits. *J. Food Biochem.* 14 421–433. 10.1111/j.1365-313X.2011.04861.x 22098335

[B41] Loreto F. Delfine S. (2000). Emission of isoprene from salt-stressed *Eucalyptus globulus* leaves. *Plant Physiol.* 123 1605–1610. 10.1104/pp.123.4.1605 10938376 PMC59117

[B42] Loreto F. Schnitzler J. P. (2010). Abiotic stresses and induced BVOCs. *Trends Plant Sci.* 15 154–166. 10.1016/j.tplants.2009.12.006 20133178

[B43] MacNicol P. K. Jacobsen J. V. (2001). Regulation of alcohol dehydrogenase gene expression in barley aleurone by gibberellin and abscisic acid. *Physiol. Plant.* 111 533–539. 10.1034/j.1399-3054.2001.1110414.x 11299019

[B44] Mallikarjuna G. Mallikarjuna K. Reddy M. K. Kaul T. (2011). Expression of *OsDREB2A* transcription factor confers enhanced dehydration and salt stress tolerance in rice (*Oryza sativa* L.). *Biotechnol. Lett.* 33 1689–1697. 10.1007/s10529-011-0620-x 21528404

[B45] Manak M. S. Paul A. L. Sehnke P. C. Ferl R. J. (2002). Remote sensing of gene expression in planta: transgenic plants as monitors of exogenous stress perception in extraterrestrial environments. *Life Support Biosph. Sci.* 8 83–91. 11987307

[B46] Mao X. G. Jia D. S. Li A. Zhang H. Y. Tian S. J. Zhang X. L. (2011). Transgenic expression of *TaMYB2A* confers enhanced tolerance to multiple abiotic stresses in *Arabidopsis*. *Funct. Integr. Genomics* 11 445–465. 10.1007/s10142-011-0218-3 21472467

[B47] Marr I. L. Suryana N. Lukulay P. Marr M. I. (1995). Determination of chlorophyll a and chlorophyll-B by simultaneous multicomponent spectrophotometry. *Fresenius J. Anal. Chem.* 352 456–460. 10.1007/BF00323366

[B48] Matsui K. (2006). Green leaf volatiles: hydroperoxide lyase pathway of oxylipin metabolism. *Curr. Opin. Plant Biol.* 9 274–280. 10.1016/j.pbi.2006.03.002 16595187

[B49] Matton D. P. Constabel P. Brisson N. (1990). Alcohol dehydrogenase gene expression in potato following elicitor and stress treatment. *Plant Mol. Biol.* 14 775–783. 10.1007/BF00016510 2102855

[B50] Mikami K. Kanesaki Y. Suzuki I. Murata N. (2002). The histidine kinase Hik33 perceives osmotic stress and cold stress in *Synechocystis* sp PCC 6803. *Mol. Microbiol.* 46 905–915. 10.1046/j.1365-2958.2002.03202.x 12421299

[B51] Msanne J. Lin J. S. Stone J. M. Awada T. (2011). Characterization of abiotic stress-responsive *Arabidopsis thaliana RD29A* and *RD29B* genes and evaluation of transgenes. *Planta* 234 97–107. 10.1007/s00425-011-1387-y 21374086

[B52] Munns R. (2002). Comparative physiology of salt and water stress. *Plant Cell Environ.* 25 239–250. 10.1046/j.0016-8025.2001.00808.x 11841667

[B53] Rossmann M. G. Moras D. Olsen K. W. (1974). Chemical and biological evolution of nucleotide-binding protein. *Nature* 250 194–199. 10.1038/250194a0 4368490

[B54] Roy S. J. Negrao S. Tester M. (2014). Salt resistant crop plants. *Curr. Opin. Biotechnol.* 26 115–124. 10.1016/j.copbio.2013.12.004 24679267

[B55] Sakuma Y. Maruyama K. Osakabe Y. Qin F. Seki M. Shinozaki K. (2006a). Functional analysis of an *Arabidopsis* transcription factor, DREB2A, involved in drought-responsive gene expression. *Plant Cell* 18 1292–1309. 10.1105/tpc.105.035881 16617101 PMC1456870

[B56] Sakuma Y. Maruyama K. Qin F. Osakabe Y. Shinozaki K. Yamaguchi-Shinozaki K. (2006b). Dual function of an Arabidopsis transcription factor DREB2A in water-stress-responsive and heat-stress-responsive gene expression. *Proc. Natl. Acad. Sci. U.S.A.* 103 18822–18827. 17030801 10.1073/pnas.0605639103 PMC1693746

[B57] Salas J. J. Sanchez C. Garcia-Gonzalez D. L. Aparicio R. (2005). Impact of the suppression of lipoxygenase and hydroperoxide lyase on the quality of the green odor in green leaves. *J. Agric. Food Chem.* 53 1648–1655. 10.1021/jf040331l 15740054

[B58] Senthil-Kumar M. Hema R. Suryachandra T. R. Ramegowda H. V. Gopalakrishna R. Rama N. (2010). Functional characterization of three water deficit stress-induced genes in tobacco and *Arabidopsis*: an approach based on gene down regulation. *Plant Physiol. Biochem.* 48 35–44. 10.1016/j.plaphy.2009.09.005 19811926

[B59] Shi H. Z. Lee B. H. Wu S. J. Zhu J. K. (2003). Overexpression of a plasma membrane Na^+^/H^+^ antiporter gene improves salt tolerance in *Arabidopsis thaliana*. *Nat. Biotechnol.* 21 81–85. 10.1038/nbt766 12469134

[B60] Shiojiri K. Kishimoto K. Ozawa R. Kugimiya S. Urashimo S. Arimura G. (2006). Changing green leaf volatile biosynthesis in plants: an approach for improving plant resistance against both herbivores and pathogens. *Proc. Natl. Acad. Sci. U.S.A.* 103 16672–16676. 10.1073/pnas.0607780103 17075049 PMC1636513

[B61] Shiojiri K. Ozawa R. Matsui K. Sabelis M. W. Takabayashi J. (2012). Intermittent exposure to traces of green leaf volatiles triggers a plant response. *Sci. Rep.* 2:378. 10.1038/srep00378 22532926 PMC3334854

[B62] Shoumskaya M. A. Paithoonrangsarid K. Kanesaki Y. Los D. A. Zinchenko V. V. Tanticharoen M. (2005). Identical Hik-Rre systems are involved in perception and transduction of salt signals and hyperosmotic signals but regulate the expression of individual genes to different extents in *Synechocystis*. *J. Biol. Chem.* 280 21531–21538. 10.1074/jbc.M412174200 15805106

[B63] Sobhanian H. Razavizadeh R. Nanjo Y. Ehsanpour A. A. Jazii F. R. Motamed N. (2010). Proteome analysis of soybean leaves, hypocotyls and roots under salt stress. *Proteome Sci.* 8:19. 10.1186/1477-5956-8-19 20350314 PMC2859372

[B64] Speirs J. Lee E. Holt K. Yong-Duk K. Steele Scott N. Loveys B. (1998). Genetic manipulation of alcohol dehydrogenase levels in ripening tomato fruit affects the balance of some flavor aldehydes and alcohols. *Plant Physiol.* 117 1047–1058. 10.1104/pp.117.3.1047 9662548 PMC34921

[B65] Strommer J. (2011). The plant ADH gene family. *Plant J.* 66 128–142. 10.1111/j.1365-313X.2010.04458.x 21443628

[B66] Sun W. N. Bernard C. Van De Cotte B. Van Montagu M. Verbruggen N. (2001). *At-HSP17.6A*, encoding a small heat-shock protein in *Arabidopsis*, can enhance osmotolerance upon overexpression. *Plant J.* 27 407–415. 10.1046/j.1365-313X.2001.01107.x 11576425

[B67] Sun Y. G. Wang B. Jin S. H. Qu X. X. Li Y. J. Hou B. K. (2013). Ectopic expression of Arabidopsis glycosyltransferase *UGT85A5* enhances salt stress tolerance in tobacco. *PLOS ONE* 8:e59924. 10.1371/journal.pone.0059924 23533660 PMC3606239

[B68] Teuber M. Zimmer I. Kreuzwieser J. Ache P. Polle A. Rennenberg H. (2008). VOC emissions of Grey poplar leaves as affected by salt stress and different N sources. *Plant Biol.* 10 86–96. 10.1111/j.1438-8677.2007.00015.x 18211549

[B69] Thompson C. E. Salzano F. M. De Souza O. N. Freitas L. B. (2007). Sequence and structural aspects of the functional diversification of plant alcohol dehydrogenases. *Gene* 396 108–115. 10.1016/j.gene.2007.02.016 17433574

[B70] Thulke O. Conrath U. (1998). Salicylic acid has a dual role in the activation of defence-related genes in parsley. *Plant J.* 14 35–42. 10.1046/j.1365-313X.1998.00093.x 15494053

[B71] Tingey D. T. Manning M. Grothaus L. C. Burns W. F. (1980). Influence of light and temperature on monoterpene emission rates from slash pine. *Plant Physiol.* 65 797–801. 10.1104/pp.65.5.797 16661285 PMC440427

[B72] Turlings T. C. Loughrin J. H. Mccall P. J. Rose U. S. Lewis W. J. Tumlinson J. H. (1995). How caterpillar-damaged plants protect themselves by attracting parasitic wasps. *Proc. Natl. Acad. Sci. U.S.A.* 92 4169–4174. 10.1073/pnas.92.10.4169 7753779 PMC41905

[B73] Vallat A. Gu H. Dorn S. (2005). How rainfall, relative humidity and temperature influence volatile emissions from apple trees in situ. *Phytochemistry* 66 1540–1550. 10.1016/j.phytochem.2005.04.038 15949824

[B74] Van der Ent S. Van Hulten M. Pozo M. J. Czechowski T. Udvardi M. K. Pieterse C. M. J. (2009). Priming of plant innate immunity by rhizobacteria and beta-aminobutyric acid: differences and similarities in regulation. *New Phytol.* 183 419–431. 10.1111/j.1469-8137.2009.02851.x 19413686

[B75] van Wees S. C. M. Luijendijk M. Smoorenburg I. Van Loon L. C. Pieterse C. M. J. (1999). Rhizobacteria-mediated induced systemic resistance (ISR) in Arabidopsis is not associated with a direct effect on expression of known defense-related genes but stimulates the expression of the jasmonate-inducible gene Atvsp upon challenge. *Plant Mol. Biol.* 41 537–549. 10.1023/A:1006319216982 10608663

[B76] Vidal R. Lopez-Maury L. Guerrero M. G. Florencio F. J. (2009). Characterization of an alcohol dehydrogenase from the Cyanobacterium *Synechocystis* sp. strain PCC 6803 that responds to environmental stress conditions via the Hik34-Rre1 two-component system. *J. Bacteriol.* 191 4383–4391. 10.1128/JB.00183-09 19411329 PMC2698509

[B77] Wei J. Kang L. (2011). Roles of (Z)-3-hexenol in plant-insect interactions. *Plant Signal. Behav.* 6 369–371. 10.4161/psb.6.3.14452 21346418 PMC3142417

[B78] Winicov I. (1998). New molecular approaches to improving salt tolerance in crop plants. *Ann. Bot.* 82 703–710. 10.1006/anbo.1998.0731

[B79] Yamauchi Y. Kunishima M. Mizutani M. Sugimoto Y. (2015). Reactive short-chain leaf volatiles act as powerful inducers of abiotic stress-related gene expression. *Sci. Rep.* 5:8030. 10.1038/srep08030 25619826 PMC4306126

[B80] Yang W. Liu X. D. Chi X. J. Wu C. A. Li Y. Z. Song L. L. (2011). Dwarf apple MbDREB1 enhances plant tolerance to low temperature, drought, and salt stress via both ABA-dependent and ABA-independent pathways. *Planta* 233 219–229. 10.1007/s00425-010-1279-6 20967459

[B81] Zhang F. Zhu G. Z. Du L. Shang X. G. Cheng C. Z. Yang B. (2016). Genetic regulation of salt stress tolerance revealed by RNA-Seq in cotton diploid wild species. *Gossypium davidsonii. Sci. Rep.* 6:620582. 10.1038/srep20582 26838812 PMC4738326

[B82] Zhou J. Li F. Wang J. L. Ma Y. Chong K. Xu Y. Y. (2009). Basic helix-loop-helix transcription factor from wild rice (OrbHLH2) improves tolerance to salt- and osmotic stress in *Arabidopsis*. *J. Plant Physiol.* 166 1296–1306. 10.1016/j.jplph.2009.02.007 19324458

[B83] Zimmerli L. Jakab C. Metraux J. P. Mauch-Mani B. (2000). Potentiation of pathogen-specific defense mechanisms in *Arabidopsis* by beta-aminobutyric acid. *Proc. Natl. Acad. Sci. U.S.A.* 97 12920–12925. 10.1073/pnas.230416897 11058166 PMC18865

