# Supplementary figures and images for: An Alcohol Dehydrogenase Gene from Synechocystis sp. Confers Salt Tolerance in Transgenic Tobacco
Source: Front Plant Sci. 2017 Nov 17;8:1965. doi: 10.3389/fpls.2017.01965 (PMC5698875; doi:10.3389/fpls.2017.01965)

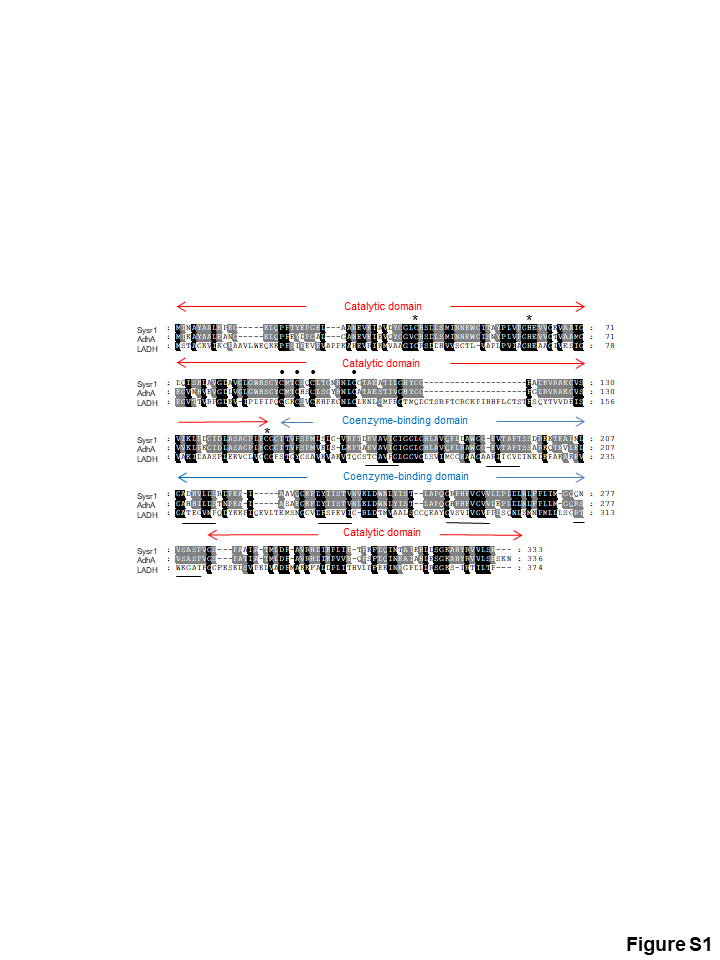

Supplement: Supplementary file 1 [file Image_1.TIF]

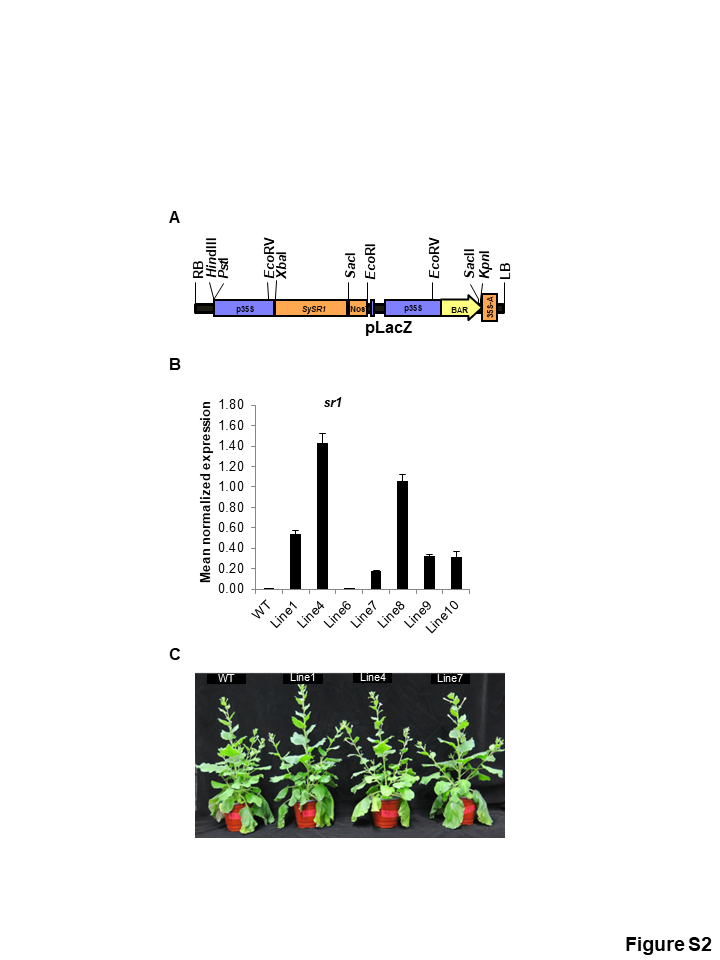

Supplement: Supplementary file 2 [file Image_2.TIF]

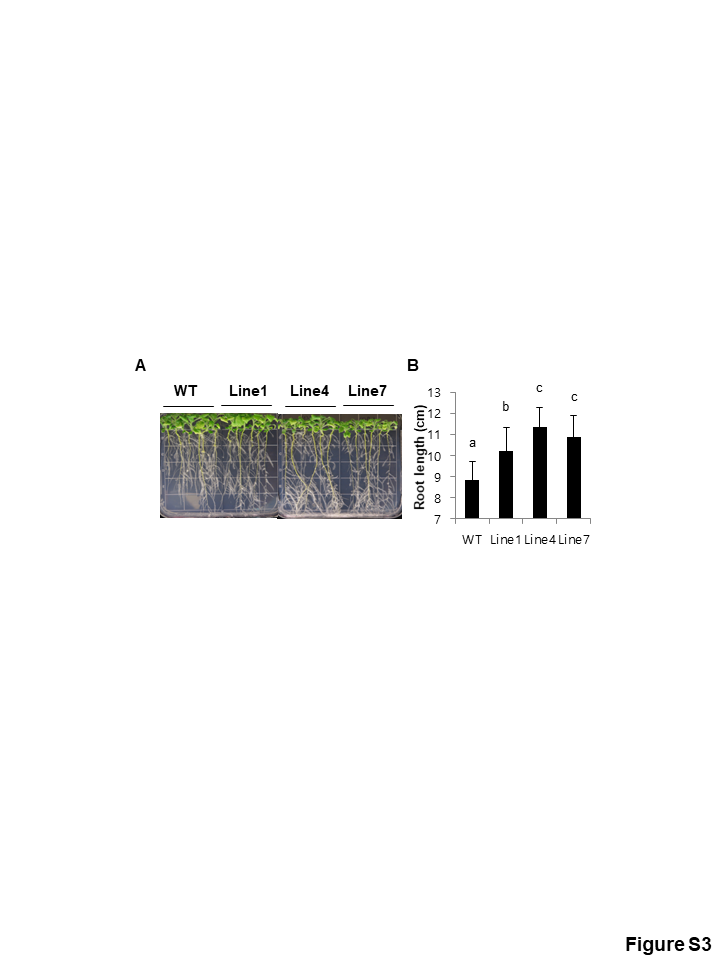

Supplement: Supplementary file 3 [file Image_3.TIF]
