# Supplementary material for: An Alcohol Dehydrogenase Gene from Synechocystis sp. Confers Salt Tolerance in Transgenic Tobacco
Source: Front Plant Sci. 2017 Nov 17;8:1965. doi: 10.3389/fpls.2017.01965 (PMC5698875; doi:10.3389/fpls.2017.01965)
Supplement: Supplementary file 4 [file Table_1.DOCX]

Table S1. Details regarding the primers used for the qRT-PCR analysis.

| Primer name | Primer sequence (5’🡪3’) | Reference or  GenBank Accession |
| --- | --- | --- |
| *sysr1*-F  *sysr1*-R | AAGTGGAAATTGCCGTGGAC  TGCAATAACCGGAATGCCAC | KY014075 |
| *NtDREB2A-*F  *NtDREB2A-*R | CAAAGGACATGGGGAAAATG  TGCCCAAGAAAGAATCATCC | ([Du *et al.*, 2013](#_ENREF_2)) |
| *HSP17.6-*F  *HSP17.6-*R | ATGTCTCTGATTCCGAGCTT  GGCGAAAGCAGAGGTTGGAAG | KJ719262 |
| *HPL-*F  *HPL-*R | CACTTAGACTTAGTCCACCTGTGC  AACACAAACTTTTCAGGATTATCA | ([Allmann *et al.*, 2010](#_ENREF_1)) |
| *RD29B-*F  *RD29B-*R | TAAGCCTGGAGAGGAGGACA  CCTTCGCCAACTTGAGTTTC | ([Du *et al.*, 2013](#_ENREF_2)) |
| *EF1α-*F  *EF1α-*R | TGAGATGCACCACGAAGCTC  CCAACATTGTCACCAGGAAGTG | ([Schmidt & Delaney, 2010](#_ENREF_3)) |

**Allmann S, Halitschke R, Schuurink RC, Baldwin IT. 2010.** Oxylipin channelling in Nicotiana attenuata: lipoxygenase 2 supplies substrates for green leaf volatile production. *Plant Cell and Environment* **33**(12): 2028-2040.

**Du XM, Zhao XL, Li XJ, Guo CJ, Lu WJ, Gu JT, Xiao K. 2013.** Overexpression of TaSRK2C1, a Wheat SNF1-Related Protein Kinase 2 Gene, Increases Tolerance to Dehydration, Salt, and Low Temperature in Transgenic Tobacco. *Plant Molecular Biology Reporter* **31**(4): 810-821.

**Schmidt GW, Delaney SK. 2010.** Stable internal reference genes for normalization of real-time RT-PCR in tobacco (Nicotiana tabacum) during development and abiotic stress. *Molecular Genetics and Genomics* **283**(3): 233-241.
